# Comparison of Concentration- and Homology-Dependent Effects of the Proinflammatory Cytokine Interleukin-1β (IL-1β) in a Bovine Chondrocyte Inflammation Model

**DOI:** 10.3390/cells14010030

**Published:** 2024-12-31

**Authors:** Robert Ossendorff, Sarah Kurth, Su Wang, Max Jaenisch, Elio Assaf, Sebastian Scheidt, Kristian Welle, Christof Burger, Dieter C. Wirtz, Andreas C. Strauss, Frank A. Schildberg

**Affiliations:** Department of Orthopedics and Trauma Surgery, University Hospital Bonn, 53127 Bonn, Germany

**Keywords:** IL-1β, inflammation model, cartilage, chondrocyte, cytokine, bovine

## Abstract

Inflammation models with the proinflammatory cytokine interleukin-1β (IL-1β) are widely used in the in vitro investigation of new therapeutic approaches for osteoarthritis (OA). The aim of this study was to systematically analyze the influence of IL-1β in a 3D chondral pellet culture model. Bovine articular chondrocytes were cultured to passage 3 and then placed in pellet culture. Titration of IL-1β (100–0.1 ng/mL) was performed with both human and bovine recombinant protein in chondrocyte culture for 2 weeks. Gene expression of anabolic (collagen 2, aggrecan, cartilage oligomeric protein (COMP), proteoglycan-4 (PRG-4)), catabolic matrix metallo proteinases (MMP-3, MMP-13), dedifferentiation (collagen 1) markers and inflammatory cytokines IL-6 and IL-8 was determined. Analysis of the cell culture medium was performed for the inflammatory markers IL-6 and nitric oxide (NO). In general, the influence of IL-1β was shown by a decrease in the expression of anabolic markers (collagen 2, aggrecan, PRG-4), whereas the catabolic markers MMP-3 and MMP-13 as well as the inflammatory markers IL-6 and IL-8 were significantly increased. This was observed both at the early time point (day 4) and at the late time point (day 14). The described inflammatory effects were confirmed by increased concentration-dependent release of NO and IL-6. The threshold concentration for a detectable effect compared to control differed between groups, but was reached earlier by homologous application of IL-1β. This study provides a systematic evaluation of IL-1β-specific effects on chondrocytes in a 3D pellet culture model, which is highly relevant for comparisons of studies in OA-specific drug development.

## 1. Introduction

Osteoarthritis (OA) is a major degenerative disease of the articular joints and affects a large portion of the population worldwide with increasing prevalence [1,2]. To date, the pathomechanism of OA is still unclear and a disease-modifying therapy is not available [3]. Articular cartilage consists of chondrocytes as single cell type, embedded in a complex extracellular matrix, which is adapted to the specific function to withstand mechanical forces in joint movement. The tissue has a low intrinsic regeneration potential and can be affected by disturbed surroundings, which can be a consequence of traumatic (osteo-) chondral lesions, joint instability (e.g., in knee ACL rupture, meniscus tear) or metabolic dysbalance [4,5]. The metabolic activity of articular chondrocytes enables a homeostasis of anabolic and catabolic factors. Extracellular matrix components collagen 2, proteoglycans (e.g., aggrecan, proteoglycan-4), and glycoproteins, including cartilage oligomeric protein (COMP), play an important role in cartilage integrity. On the other hand, catabolic enzymes such as matrix metallo proteinases (MMP-3, MMP-13) contribute to a cleavage of ECM components and a permanent cartilage turnover, which is adapted to the mechanical load in an equilibrium. OA leads to disturbed homeostasis with increased catabolic processes and decreased anabolic factors [6,7].

The role of inflammation in the pathophysiology of OA is an increasingly upcoming topic of interest. The proinflammatory cytokine IL-1β plays a key role in the pathomechanism of inflammatory joint diseases such as rheumatoid arthritis and OA and is discussed as a marker of disease progression [8,9,10]. Elevated IL-1β concentration in the synovial fluid leads to an induction of inflammatory processes including nitric oxide synthase activation, interleukin-6 (IL-6), and interleukin-8 (IL-8) production. Consequently, in vitro chondrocyte inflammation models with IL-1β are often used for systematical analysis of potential drug candidates for OA [11,12,13]. IL-1β is supplemented to the medium of 2D and 3D chondrocyte in-vitro culture to simulate inflammatory surroundings. However, available studies display a high heterogeneity with regard to concentration and origin (homology) of the recombinantly produced cytokine and a lack of systematic reports of model characteristics. The aim of this study was to systematically analyze the influence of IL-1β in a standardized 3D chondrocyte pellet culture with different concentrations and to analyze the difference in the efficacy of cytokine homology to the used model.

## 2. Materials and Methods

### 2.1. Experimental Design

**Figure 1:**
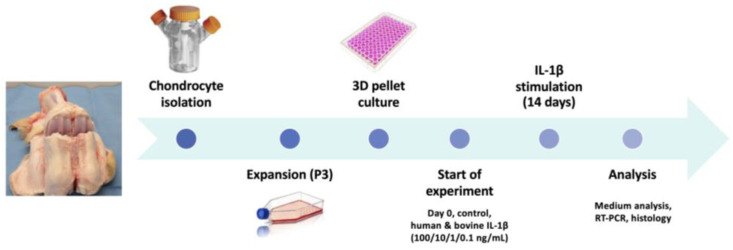
Schematical overview of the experimental design. Chondrocytes were isolated from bovine fetlock joints, expanded to passage 3 and transferred in 3D pellet culture for chondrogenic differentiation. Cytokine supplementation with interleukin-1β (IL-1β) was performed over a period of 14 days in different concentrations (range 0.1–100 ng/mL). Analysis was performed on gene expression level with RT-PCR as well as utilizing biochemical and histological analyses to evaluate the specific influence on inflammation and chondrocyte differentiation.

Cartilage from the fetlock joints of healthy bovine subjects aged 6–24 months was collected (Figure 1). The animals were euthanized on the same day by a local butcher for food production, therefore, ethical approval was not required for this process. In this standardized model, chondrocytes from the articular cartilage were isolated and expanded up to passage 3 (5–8 population doublings) as published previously [14,15,16]. All samples were cultured for one week. Afterwards, IL-1β was supplemented to simulate an inflammatory environment, with the start of cytokine stimulation defined as day 0. Either human or bovine IL-1β was used in the concentrations 100 ng/mL, 10 ng/mL, 1 ng/mL, and 0.1 ng/mL. The medium was changed three times per week. The experiment involved four different donors and was concluded on day 4 for gene expression analysis and day 14 for gene expression and protein analysis of the resulting tissue for all groups.

### 2.2. Isolation and Culture of Articular Chondrocytes

The cartilage fragments, each smaller than 10 mm^3^, underwent pre-digestion using 0.1% pronase (Merck, Darmstadt, Germany), followed by a 14-h digestion in collagenase 2 (Worthington, Lakewood, NJ, USA). Chondrocytes were initially seeded at a density of 16.7 × 10^3^ cells/cm^2^ in Dulbecco’s modified Eagle medium (DMEM high glucose) (Thermo Fisher Scientific, Waltham, MA, USA) with 10% fetal bovine serum (FBS) (Bio & Sell, Feucht, Germany). Upon reaching 90% confluency, cells were passaged by undergoing pre-digestion with collagenase 2 (30 min) for cleavage of trypsin-resistant extracellular components (e.g., collagens) and trypsin digestion (5–20 min), then re-seeded at the same density as previously described [14]. The medium change was performed every other day. At passage 3, cells were transferred to pellet culture as previously described [14]. In brief, 0.25 million chondrocytes per well were placed in 96-well V-bottom non-adhesive plates (Greiner, Kremsmünster, Austria). The plates were centrifuged at 500× *g* for 5 min to form chondrocyte pellets and were cultivated for one week under free swelling conditions using a chondropermissive culture medium.

### 2.3. Chondrocyte Inflammation Model

Human recombinant IL-1β (PeproTech, Cranbury, NJ, USA) or bovine recombinant IL-1β (Thermo Fisher Scientific, Waltham, MA, USA) were supplemented to the medium. Human IL-1β was a recombinant protein from E. coli with a molecular weight of 17.3 kDa and 153 amino acids. Bovine IL-1β was a recombinant protein from yeast species *Pichia pastoris*. The molecular weight was 17.7 Da from 114–266 amino acids. Different concentrations were selected in the range of previous studies including 100 ng/mL, 10 ng/mL, 1 ng/mL, 0.1 ng/mL [17,18,19,20,21,22]. We selected the range according to the available literature to compare the effects of different cytokine concentrations with a 10-fold difference between the concentrations to cover the whole range of currently used concentrations of the IL-1β inflammation model. Medium change was performed six times over 14 days and supernatants were collected for further biochemical analysis.

### 2.4. Interleukin-6 Enzyme Linked Immunosorbent Assay (IL-6 ELISA)

The medium was analyzed at various time points (0, 2, 4, 6, 8, 12, 14 days) of 4 independent biological replicates of each treatment group to determine IL-6 concentration using a bovine IL-6 ELISA assay kit (Kingfisher Biotech, St. Paul, MN, USA) as described elsewhere [23]. The Bovine IL-6 Do-It-Yourself ELISA contained a capture antibody (Anti-Bovine IL-6 Polyclonal Antibody), standard (Bovine IL-6 Recombinant Protein), and detection antibody (Biotinylated Anti-Bovine IL-6 Polyclonal Antibody). IL-6 plays an important role in the chondrocyte-related inflammatory response to external IL-1β stimulation [17].

### 2.5. Nitric Oxide Analysis

The medium was analyzed at all separate time points for nitric oxide (NO) content using a Griess diazotization reaction assay against the nitrite standard (Promega, Walldorf, Germany). Nitric oxide is an unspecific inflammation marker, which is used to monitor the inflammatory response in chondrocyte inflammation models [24].

### 2.6. Glycosamioglycan Quantification

Cell pellets were digested by proteinase K for further biochemical analysis (0.5 mg/mL; Roche, Basel, Switzerland) in phosphate buffer overnight. The samples were analyzed for glycosaminoglycan (GAG) content using a 1,9-dimethyl-methylene blue (DMMB; Sigma-Aldrich, St. Louis, MO, USA) dye-binding assay against the standard bovine chondroitin sulfate (Sigma-Aldrich). The DMMB assay detects all sulfated glycosaminoglycans including chondroitin sulfates (CS), keratan sulfates (KS), and heparan sulfates (HS) as central components of the extracellular matrix. Previous studies reported a reduced GAG synthesis by IL-1β stimulation of articular chondrocytes [18].

### 2.7. RNA Extraction, Reverse Transcription, and Gene Expression Analysis

All samples were homogenized using a tissue-lyzer system (Qiagen, Hilden, Germany) in 1 mL of trizol reagents (TRI reagents, Molecular Research Center, Cincinnati, OH, USA) for 10 min at 30 Hz. Ribonucleic acid (RNA) was extracted using a precipitation method employing bromochloropropane (BCP, Sigma-Aldrich) in a 1:10 volume ratio for phase separation. RNA cleanup was performed using a column-based extraction kit specific to tissues (Qiagen). Reverse transcription was carried out using TaqMan reverse transcription reagents (Applied Biosciences) with 1 µg of total RNA to synthesize complementary DNA (cDNA). Gene expression analysis was conducted using a TaqMan Real-Time polymerase chain reaction (PCR) system (Thermo Fisher Scientific) in TaqMan master mix, employing bovine TaqMan Assays on demand (Thermo Fisher Scientific) targeting specific markers such as chondrogenic markers collagen 2 (COL2A1, Bt03251861_m1), aggrecan (ACAN, Bt03212186_m1), cartilage oligomeric protein (COMP, Bt04299192_g1), collagen 1 (COL1A2, Bt03214860_m1) as marker for dedifferentiation, matrix metalloproteinases MMP-3 (Bt04259490_m1), and MMP-13 (Bt03214050_m1) associated with catabolic processes, as well as cytokines IL-6 (Bt03211905_m1) and IL-8 (CXCL8, Bt03211906_m1). To quantify gene expression, it was normalized to the endogenous control 18S ribosomal RNA. A comparative analysis was conducted using threshold cycle (CT) values normalized to the mean CT values of 18S (ΔCT) and then further normalized to day 0 (ΔΔCT). Relative messenger RNA (mRNA) expression levels were calculated using the 2-ΔΔCT method.

### 2.8. Histology

The chondrocyte pellet samples were initially fixed in 70% methanol and subsequently underwent paraffin fixation using a carousel tissue processor. This paraffin fixation process spanned 24 h and included steps in 70% ethanol, 96% ethanol, 100% ethanol, xylene, and paraffin. The paraffin-embedded samples were then sliced into 5 µm sections using a microtome. For assessment of cell morphology and extracellular matrix deposition, staining with safranin O and Fast Green (Sigma-Aldrich) was carried out. Paraffin sections were first stained with Weigert’s Iron Hematoxylin Stain Kit (Sigma-Aldrich) for 10 min, followed by 0.02% Fast Green (Sigma-Aldrich) in ultrapure (ddH_2_O) water for 6 min and 0.1% safranin O for 10 min. A collection of images was taken using an Olympus IX81 inverted fluorescence microscopy and the CellSens Dimension software version 4.2 (Olympus Corporation, Hamburg, Germany). Semi-quantitative analysis was used to examine differences between groups. Proteoglycans are a main component of the extracellular matrix and affected in an early disease stage of osteoarthritis [25].

### 2.9. Immunohistochemistry of Anti-Active Caspase 3

Apoptosis was evaluated by immunolabeling of anti-active caspase 3 in a the Vectastain Elite ABC Kit (Vector Laboratories, Newark, CA, USA) with the ABC-based (avidin-biotin-complex) indirect staining method as previously described [15]. The primary antibody anti-active caspase 3 (Promega) rabbit polyclonal, diluted 1:200 with PBS-Tween and 1:200 goat serum (Vector Laboratories) was incubated in an overnight stain. As a second antibody, the biotinylated anti-rabbit IgG (H1L; Vector Laboratories) was diluted 1:200. After avidin-biotin-peroxidase complex building DAB (3,3’-diaminobenzidine) was added, which stained the specifically attached target antigen by multiple peroxidase enzyme molecules. Mayer hematoxylin (Fluka) was used as counterstain.

### 2.10. Statistics

Statistical analysis was conducted using Statistical Package für Social Sciences (SPSS v 24; IBM, Armonk, NY, USA). The Kolmogorov-Smirnov test was utilized to assess normal distribution. To test for significant differences among the groups as independent variables and the analytical results as dependent groups, a non-parametric Wilcoxon-Mann-Whitney test was applied. Statistical significance was set at *p* < 0.05. Graphical representation of the data was created using Graphpad Prism 9 (Graphpad Software Inc., San Diego, CA, USA).

## 3. Results

### 3.1. Anabolic Markers

**Figure 2:**
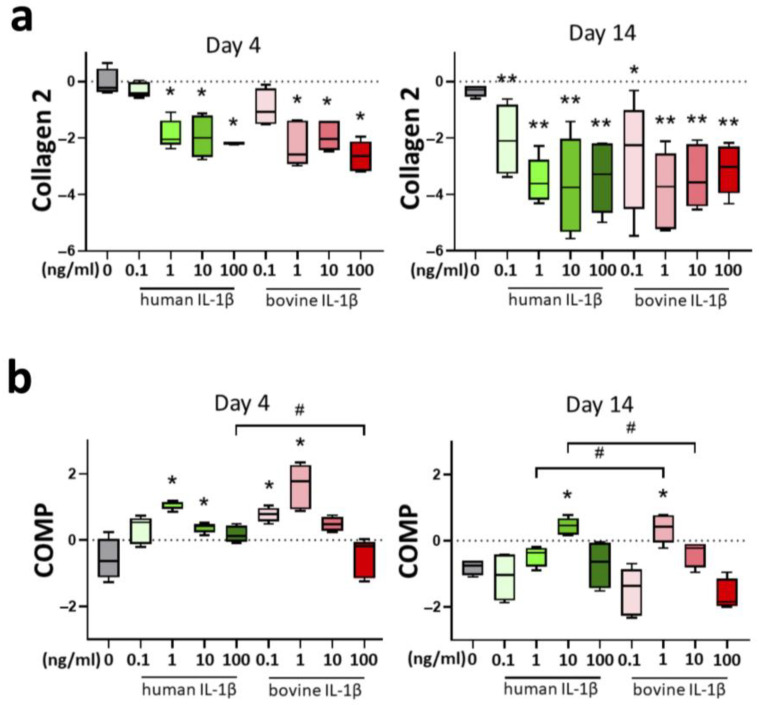
Influence of IL-1β supplementation in a chondrocyte inflammation model with different concentrations (0.1–100 ng/mL) and origin (human/bovine) on mRNA levels of anabolic markers (**a**) collagen 2, and (**b**) cartilage oligomeric protein (COMP) relative to day 0. Control is shown in grey without cytokine supplementation. Results were transformed by natural logarithm and visualized in box plots. Statistical analyses were performed between untreated (0 ng/mL) and treated samples (indicated by asterisk, * *p* < 0.05, ** *p* < 0.01) and between human and bovine samples (indicated by hashtag, ^#^ *p* < 0.05). IL-1β: interleukin-1β.

In general, supplementation of the proinflammatory cytokine IL-1β resulted in a decrease in anabolic markers in chondrocytes at the gene expression level in the short-term (4 days) and long-term (14 days). Collagen 2 (Figure 2a), as main component of the extracellular matrix, showed a cytokine concentration-dependent downregulation on day 4 with a threshold of 1 ng/mL. Fourteen days of IL-1β stimulation resulted in a decrease of collagen 2 expression in all groups compared to control. There was no significant difference in the cytokine efficacy between human and bovine IL-1β.

Cartilage oligomeric protein (COMP) is an important component for the fibrillogenesis of collagen and a marker for cartilage turnover. Chondrocyte gene expression of COMP (Figure 2b) was stimulated by IL-1β supplementation at day 4 at the concentration of 1 and 10 ng/mL for human IL-1β, and 0.1 and 1 ng/mL for bovine IL-1β. Interestingly, the long-term culture (14 days) showed a similar pattern with significant upregulation of COMP expression in 10 ng/mL for bovine IL-1β and 1 ng/mL for human IL-1β.

**Figure 3:**
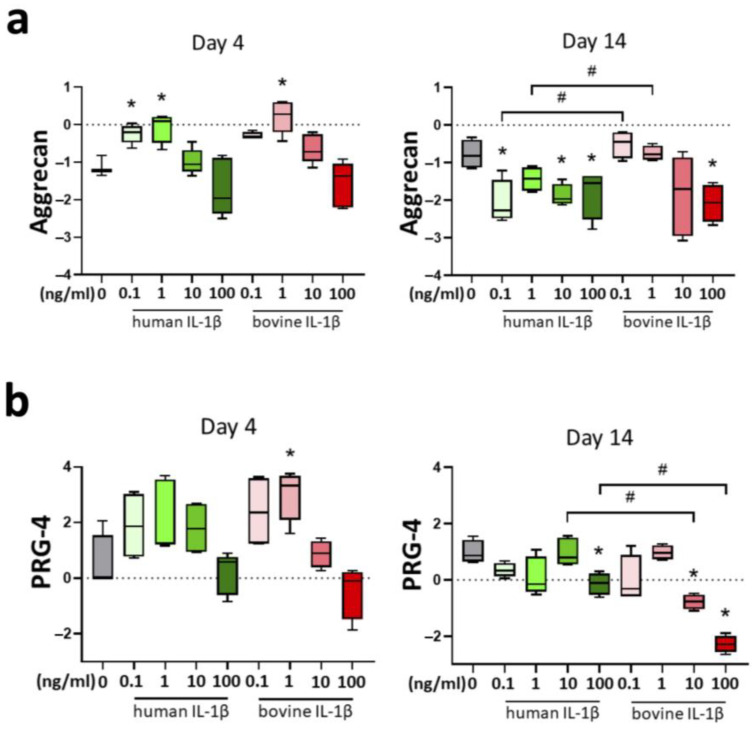
Influence of IL-1β supplementation in a chondrocyte inflammation model with different concentrations (0.1–100 ng/mL) and origin (human/bovine) on mRNA levels of anabolic markers (**a**) aggrecan and (**b**) proteoglycan-4 (PRG-4) relative to day 0. Control is shown in grey without cytokine supplementation. Results were transformed by natural logarithm and visualized in box plots. Statistical analyses were performed between untreated (0 ng/mL) and treated samples (indicated by asterisk, * *p* < 0.05) and between human and bovine samples (indicated by hashtag, ^#^ *p* < 0.05). IL-1β: interleukin-1β.

Aggrecan is a major proteoglycan of the extracellular matrix of articular cartilage and is important for the physiological function of load absorption. There was a significant upregulation in chondrocyte gene expression of aggrecan after 4 days stimulation with IL-1β for the concentrations 0.1 and 1 ng/mL (human IL-1β) and 1 ng/mL (bovine IL-1β) (Figure 3a). Interestingly, day 14 showed a contrary pattern with downregulation of aggrecan at concentrations 0.1, 10, 100 ng/mL with human IL-1β and 100 ng/mL with bovine IL-1β. The effects were significantly different between human and bovine IL-1β supplementation.

Proteoglycan-4 (PRG-4) as a lubricant can predominantly be found in the surface zone of articular cartilage and the synovial fluid. PRG-4 showed a heterogenous chondrocyte gene expression pattern at day 4, with an upregulation in low concentration, with only significant difference in 1 ng/mL bovine IL-1β compared to control (Figure 3b). Fourteen days cytokine stimulation resulted in downregulation of PRG-4 in bovine chondrocytes at a concentration of 100 ng/mL (human and bovine IL-1β). Interestingly, 10 ng/mL bovine IL-1β significantly lowered PRG-4 expression compared to control and compared to human IL-1β.

**Figure 4:**
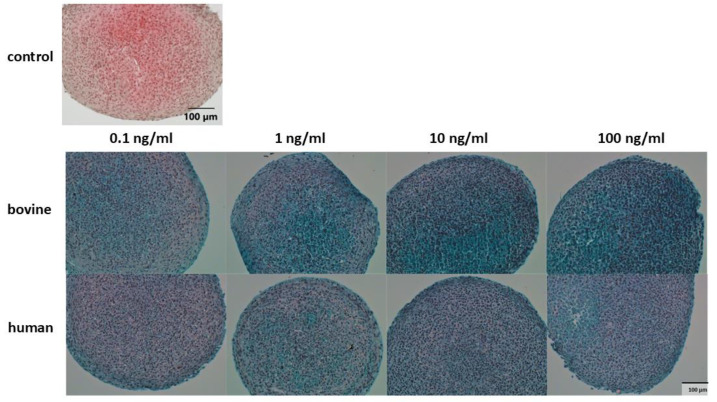
Safranin O/Fast Green stain of P3 chondrocytes in a chondrocyte inflammation model using IL-1β with different concentrations (0.1–100 ng/mL) and origin (human/bovine). Control was untreated without cytokine stimulation. Scale bar 100 µm. IL-1β: interleukin-1β.

**Figure 5:**
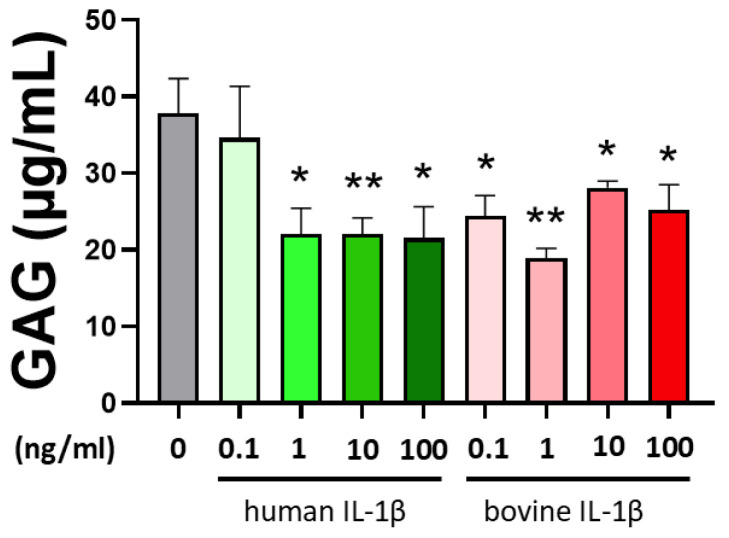
Quantitative analysis of sulfated glycosaminoglycan (GAG) retention in a 3D pellet inflammation model with bovine passage 3 chondrocytes using IL-1β with different concentrations (0.1–100 ng/mL) and origin (human/bovine). Control is shown in grey without cytokine supplementation. The results are presented as the mean + SEM from three different donors. Statistical analyses were performed between untreated (0 ng/mL) and treated samples (indicated by asterisk, * *p* < 0.05, ** *p* < 0.01) and between human and bovine samples (no detected significance in this case). IL-1β: interleukin-1β.

Histological analysis was performed using safranin O Fast Green stain method to evaluate regenerate quality. In general, the amount of proteoglycans was lower compared to control (Figure 4). There was no difference between the different concentrations and cytokine species, which was confirmed in quantification of the sulfated glycosaminoglycans (Figure 5). The GAG retention in the chondrocyte pellet was significantly lower in the presence of IL-1β for all bovine cytokine groups. Interestingly, supplementation of 0.1ng/mL human IL-1β did not show a significant influence on GAG synthesis.

### 3.2. Catabolic Markers

**Figure 6:**
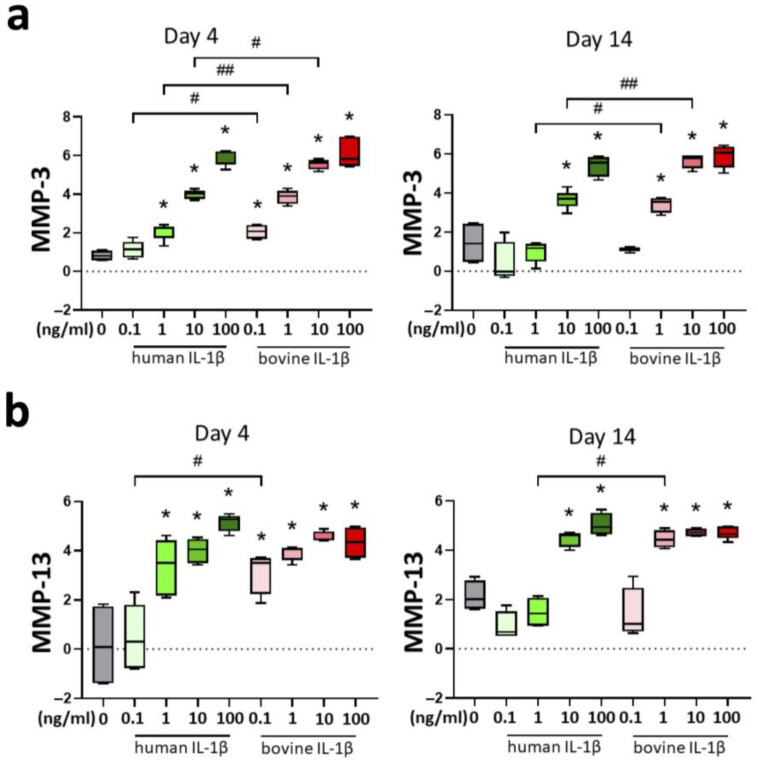
Influence of IL-1β supplementation in a chondrocyte inflammation model with different concentrations (0.1–100 ng/mL) and origin (human/bovine) on mRNA levels of catabolic markers (**a**) matrixmetalloproteinase-3 (MMP-3), and (**b**) matrixmetalloproteinase-13 (MMP-13) relative to day 0. Control is shown in grey without cytokine supplementation. Results were transformed by natural logarithm and visualized in box plots. Statistical analyses were performed between untreated (0 ng/mL) and treated samples (indicated by asterisk, * *p* < 0.05) and between human and bovine samples (indicated by hashtag, ^#^ *p* < 0.05, ^##^ *p* < 0.01). IL-1β: interleukin-1β.

Matrix metallo proteinases MMP-3 and MMP-13 were analyzed for the evaluation of catabolic turnover in the chondrocyte inflammation model. These enzymes cleave components of the ECM and are discussed as an important marker of OA and disease progression. IL-1β supplementation increased gene expression of MMP-3 with a threshold of 1 ng/mL for human and 0.1 ng/mL for bovine cytokine in a concentration-dependent pattern at day 4 (Figure 6a). The effects were significantly stronger with bovine IL-1β compared to human IL-1β. A similar pattern was detected at day 14 with a threshold of 10 ng/mL for human and 1 ng/mL for bovine IL-1β resulting in significant differences compared to control.

Gene expression of MMP-13 was also elevated by supplementation of IL-1β with concentration-dependent differences (Figure 6b). Higher cytokine concentration resulted in stronger effects with a threshold of 1 ng/mL for human and 0.1 ng/mL for bovine IL-1β for day 4. Long-term culture over 14 days resulted in significant upregulation of MMP-13 compared to control with a threshold of 10 ng/mL for human and 1 ng/mL for bovine IL-1β.

### 3.3. Inflammation

**Figure 7:**
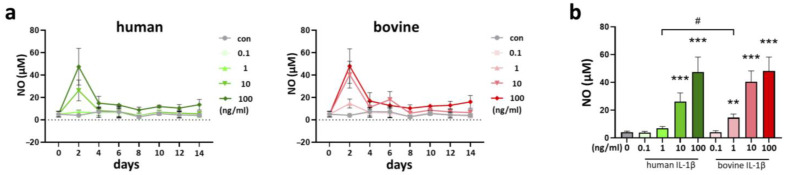
(**a**) Dynamic evaluation of nitric oxide (NO) release (µM) and (**b**) cumulative analysis of NO release into the medium (µM) over 14 days of culture with supplementation of human and bovine IL-1β in different concentrations (0.1–100 ng/mL) in a chondrocyte pellet culture. Statistical analyses were performed between untreated (0 ng/mL) and treated samples (indicated by asterisk, ** *p* < 0.01, *** *p* < 0.001) and between human and bovine samples (indicated by hashtag, ^#^ *p* < 0.05). IL-1β: interleukin-1β.

The inflammation profile of the chondrocyte pellet culture model with IL-1β was evaluated by the analysis of nitric oxide (NO) release into the medium over 14 days of culture. Interestingly, highest peak concentrations of NO were detected on day 2 for human and bovine IL-1β stimulation (Figure 7a).

Cumulative analysis of NO release into the medium over 14 days culture showed cytokine concentration-dependent effects (Figure 7b). Homologous use of IL-1β lead to a stronger release of NO into the medium compared to human IL-1β. The threshold to induce a significant cytokine response was 1 ng/mL for bovine IL-1β, whereas it was 10 ng/mL for human IL-1β.

**Figure 8:**
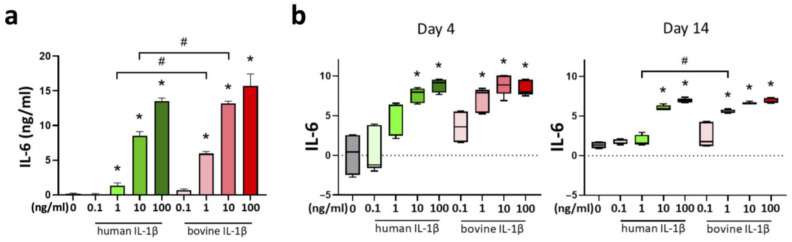
(**a**) Cumulative evaluation of IL-6 release (ng/mL) into the medium over 14 days of culture and mRNA levels at indicated time points for (**b**) IL-6 in a chondrocyte inflammation model with IL-1β in different concentrations (0.1–100 ng/mL) and origins (human/bovine). Control is shown in grey without cytokine supplementation. Results were transformed by natural logarithm and visualized in box plots. Statistical analyses were performed between untreated (0 ng/mL) and treated samples (indicated by asterisk, * *p* < 0.05) and between human and bovine samples (indicated by hashtag, ^#^ *p* < 0.05). IL-1β: interleukin-1β, IL-6: interleukin-6.

The release of the proinflammatory cytokine Interleukin-6 (IL-6) was increased by supplementation of IL-1β. This effect was concentration-dependent with a threshold concentration of 1 ng/mL for human and bovine IL-1β (Figure 8a). A similar pattern was detected in the chondrocyte gene expression analysis of IL-6 at day 4 and 14 (Figure 8b). Furthermore, the effects were stronger by using bovine IL-1β compared to human IL-1β for the concentrations 1 ng/mL and 10 ng/mL IL-1β.

**Figure 9:**
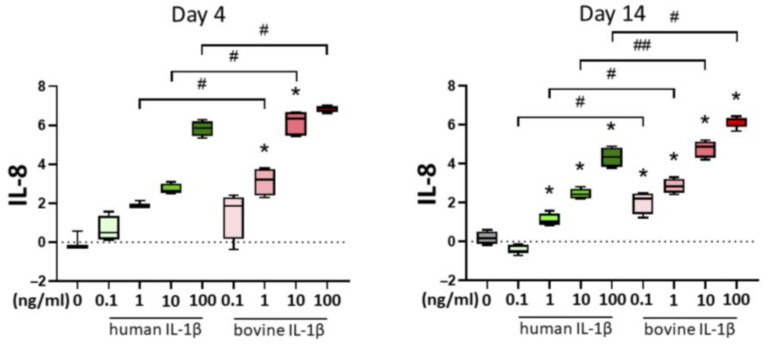
Gene expression analysis of IL-8 in a 3D pellet chondrocyte inflammation model with supplementation of IL-1β. Results are transformed by natural logarithm and visualized in box plots. Control is shown in grey without cytokine supplementation. Statistical analyses were performed between untreated (0 ng/mL) and treated samples (indicated by asterisk, * *p* < 0.05) and between human and bovine samples (indicated by hashtag, ^#^ *p* < 0.05, ^##^ *p* < 0.01). IL-1β: interleukin-1β; IL-8: interleukin-8.

The inflammation marker interleukin-8 (IL-8) is well-known as an inflammatory mediator. Gene expression analysis of IL-8 showed a concentration-dependent pattern in the chondrocyte inflammation model (Figure 9). Higher IL-1β concentration increased the effect. After 4 days of cytokine stimulation, strongest expression was detected in the groups stimulated with bovine IL-1β compared to human IL-1β with a threshold of 1 ng/mL. Long-term cytokine stimulation over 14 days showed strong concentration-dependent and homology-specific effects with stronger effects in higher IL-1β concentrations and stronger expression of IL-8 by supplementation of bovine IL-1β compared to human IL-1β. The differences between bovine and human IL-1β were significant at day 4 for 1–100 ng/L and at day 14 from 0.1–100 ng/mL.

### 3.4. Apoptosis

**Figure 10:**
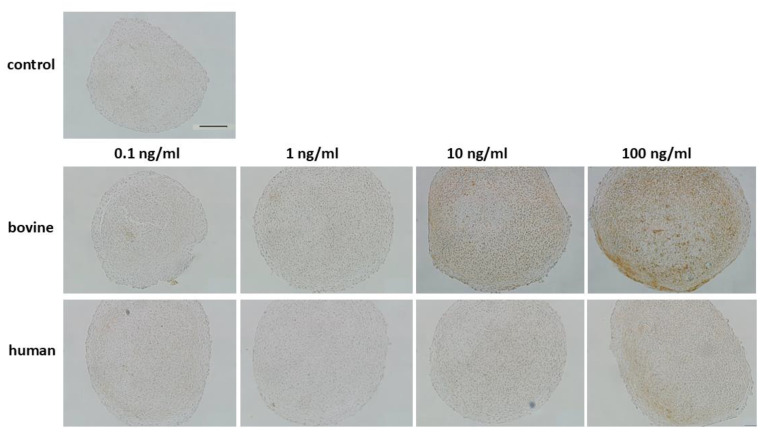
Caspase 3 immunohistochemistry of bovine passage 3 chondrocyte inflammation model with supplementation of IL-1β with different concentrations (0.1–100 ng/mL) and origin (human/bovine). Brown intracellular stain indicates apoptotic cells. Control was untreated without cytokine stimulation. Representative images are shown. Scale bar 100 µm. IL-1β: interleukin-1β.

Immunolabeling of anti-active caspase 3 (Figure 10 and Figure S1) showed a concentration-dependent intracellular stain of IL-1β stimulated chondrocytes in a 3D pellet model. The threshold concentration of supplemented bovine IL-1β was 10 ng/mL and showed the strongest effect with 100 ng/mL. For human IL-1β, the pro-apoptotic influence of the cytokine was weaker and showed intracellular stain in pellet constructs at 100 ng/mL.

## 4. Discussion

### 4.1. Key Findings

This study investigated the influence of IL-1β on bovine chondrocytes in a standardized bovine 3D pellet model over 14 days. IL-1β showed a concentration-dependent negative influence on chondrogenesis. This effect was evident by downregulation of the chondrocyte gene expression of the anabolic markers collagen 2, aggrecan, and PRG-4. Furthermore, catabolic markers MMP-3 and MMP-13 were upregulated by supplementation of IL-1β. Inflammation markers Interleukin-6 and -8 (IL-6, IL-8) were elevated. The anti-anabolic, catabolic, proinflammatory, and pro-apoptotic influence of IL-1β was also detected on protein level in a reduced glycosaminoglycan synthesis, reduction proteoglycan stain, induced release of nitric oxide and IL-6 into the medium and intracellular stain of anti-active caspase 3. The threshold concentration for a significant influence on chondrocyte metabolism was 1 ng/mL. Homologous bovine IL-1β demonstrated stronger effects compared to human IL-1β.

### 4.2. IL-1β-Related Reduction of Anabolic Factors

In our study IL-1β showed a strong concentration-dependent influence on gene expression of the anabolic markers collagen 2, aggrecan, COMP, and PRG-4, which are all central components of the extracellular matrix and key proteins for chondrocyte homeostasis. Mohanraj et al. [26] cultivated native bovine chondrocytes (P0) and supplemented IL-1β (1–10 ng/mL) in a 3D agarose gel matrix over 6 days. They demonstrated a concentration-dependent sGAG and collagen loss. Lv et al. [18] investigated the influence of bovine recombinant IL-1β (1–25 ng/mL) in a bovine cartilage explant model during 24 days in vitro culture. They detected a strong influence of the cytokine with a threshold concentration of 1 ng/mL. After 24 days of cytokine stimulation, they detected a downregulation of aggrecan on gene expression level of bovine native chondrocytes. However, collagen 2 was not different to control. On protein level, a significant loss of sGAG was detected, but collagen quantification did not show statistical difference. These strong catabolic effects could be detected with a concentration of 1 ng/mL, which is in line with our findings. Tao et al. [21] reported in a murine chondrocyte inflammation model with homologous recombinant IL-1β (1–20 ng/mL), a cytokine dependent reduction of collagen 2 expression with a threshold concentration of 5 ng/mL to induce significant effects. Collagen 2 expression was significantly reduced compared to control in a human short-term chondrocyte inflammation model with 10 ng/mL IL-1β over 24 h [6,27]. Furthermore, Hwang et al. reported a high sGAG reduction by 1 ng/mL IL-1β supplementation in a human cartilage explant model [20].

Surprisingly, the increase of COMP was only transient in our study. One possible explanation could be that low/mild inflammation could stimulate repair in the early stages. However, this effect could also be purely due to some counter regulation of the underlying pathway. As this effect needs intensive molecular analysis to be clarified, we do not want to speculate too much, but feel that there may be some room for further detailed molecular studies.

### 4.3. Matrix Metalloproteinases (MMP-3 and -13) Are Strongly Induced by IL-1β

This study showed a strong concentration-dependent increase of the gene expression of the catabolic markers MMP-3 and MMP-13, which are responsible for ECM cleavage. The threshold concentration to induce significant higher expression was 1 ng/mL. IL-1β induced MMP-3 and MMP-13 elevation was previously reported by Lv et al. [18], who detected 189 fold increase of chondrocyte gene expression by supplementation of 1 ng/mL after 48 h culture in bovine cartilage explants and a 41 fold increase for MMP-13. Furthermore, they reported an increase of the catabolic markers ADAMTS-4 (A Disentigrin and Metalloproteinase with Thrombo Spondin Motifs 4) and ADAMTS-5, which are known to cleave aggrecan. Human chondrocytes also showed an IL-1β-induced MMP-3 and MMP-13 induction over 24 h of culture in a cytokine concentration of 1–10 ng/mL [15,17,19].

### 4.4. Inflammation Is a Central Component in the IL-1β Chondrocyte Inflammation Model

This chondrocyte inflammation model demonstrated a strong concentration-dependent induction of the inflammatory markers IL-6 and IL-8 on gene expression level, which was confirmed on protein level by the release of IL-6 into the medium. The indirect inflammation marker nitric oxide (NO) also showed an IL-1β concentration-dependent elevation. Inflammatory markers were increased with a threshold concentration of 1 ng/mL IL-1β. Mohannraj et al. [26] demonstrated in a 3D bovine chondrocyte inflammation model with IL-1β (1–10 ng/mL) over 6 days of culture, a concentration-dependent NO release into the medium with a peak concentration in the early phase (day 3), which is in line with our results. In human chondrocyte inflammation model, 10 ng/mL human IL-1β induced a NO release after 24 h with a doubling of the concentration compared to the control [19]. Moreover, IL-6 and TNFα concentration were elevated after supplementation with 1 ng/mL IL-1β compared to control. Zigon-Branc et al. [17] could detect a significantly increased IL-6 and IL-8 concentration already after 24 h of 1 ng/mL IL-1β stimulation in a 3D model with expanded chondrocytes. Fan et al. [28] reported a concentration-dependent IL-1β related IL-6 stimulation of native human osteoarthritic chondrocytes (P0) with a threshold concentration of 0.01 ng/mL after 4 days of cytokine stimulation. In our results, high concentrations (>10 ng/mL) of bovine IL-1β stimulation led to induction of apoptosis. IL-1β is well-known as pro-apoptotic cytokine [29]. The role of IL-1β activated apoptosis signaling pathways plays a central role in the development and progression of osteoarthritis [22,30]. Although we found a clear association between IL-1β and pro-apoptotic signs in our spheroid model, we strongly recommend that these effects be verified in a variety of chondrocyte cultures using other imaging and molecular techniques.

### 4.5. Standardization of Inflammation Models Is an Urgent Need for Further Studies

In this study, we used a standardized chondrocyte pellet culture model to systematically evaluate the influence of the proinflammatory cytokine IL-1β with different cytokine concentrations with homologous (bovine) and human origin. In our literature analysis, we could not find any systematic analysis of the commonly used chondrocyte inflammation model. There is a current lack of reporting of essential information for comparison to other studies including the origin of the cytokine and the applied cytokine concentration. Furthermore, there is high heterogeneity in the described in vitro models concerning (1) species, (2) cell extraction and expansion procedure, and (3) time of cytokine stimulation. All these factors can influence the outcome and limit the comparability to other studies. Especially in human models, the chondrocytes were extracted from disturbed surroundings (osteoarthritis) with different stages of the disease. Another aspect is the culture method (monolayer vs. 3D culture), which strongly influences the specific phenotype. Caron et al. [31] showed that redifferentiation of dedifferentiated human articular chondrocytes was most potent in 3D cultures, whereas a hypertrophic phenotype was achieved in monolayer 2D cultures. They detected higher gene expression level of collagen 2 and aggrecan in chondrocytes of 3D culture compared to 2D. Moreover, GAG synthesis was higher in 3D culture. In this study, we only focused on 3D culture models, which simulate the physiological conditions of articular cartilage [31,32]. Furthermore, the homology of the supplemented cytokine plays an important role for the expected cytokine related effect. This was of special importance in the inflammation model with lower concentrations of IL-1β (0.1–1 ng/mL). An explanation might be the lower affinity of human IL-1β to the bovine IL-1β receptor, which is related to a different molecular structure of the protein. This study provides a standardized evaluation of the IL-1β induced chondrocyte inflammation model.

This reference work enables us to better compare the cytokine related outcome of different studies. Most importantly, authors should report all experimental details. Here, it could be beneficial if in the future, a guideline for minimal information for studies with inflammation models would be available including key factors as the (1) species of chondrocyte or cartilage, (2) the passage number of chondrocytes, (3) concentration and specific description of the cytokine, (4) the duration of the experiment, (5) the type of intervention (including e.g., mechanical stimuli, growth factors), (6) analysis of the resulting tissue.

### 4.6. Value of Clinical Translation and Limitations of the Chondrocyte Inflammation Model

The bovine chondrocyte inflammation model with IL-1β provides a high standardization and reproducibility. For the evaluation of pathomechanisms and the therapeutic potential of new disease-modifying drugs, this model as in vitro model can be used. For further simulation of physiological surroundings, the model can be used in a joint-specific bioreactor [19]. This model focused on chondrocytes and the influence of a single cytokine (IL-1β) supplementation in vitro. The physiological conditions are complex, with a heterogeneity of different factors, including different cytokines and growth factors. On the other hand, cell communication and cell-cell interaction are important in the pathogenesis of inflammatory pathologies. All these complex factors cannot be sufficiently simulated in an in vitro model. This study used a 3D inflammation model with expanded chondrocytes (passage 3). The process of monolayer cell expansion and passaging can influence functional phenotype [16]. Other conditions with explant culture, different passages and also 2D culture need to be analyzed for the IL-1β related effect on chondrocytes in further studies. Moreover, this study focused on a 14 day chondrocyte inflammation model with IL-1β. In physiological situations, cytokine stimulation includes a longer period especially in chronic degenerative diseases (years). Therefore, in vivo models are necessary for translation into clinical application.

## 5. Conclusions

This study systematically evaluated the chondrocyte inflammation model with IL-1β and demonstrated a concentration-dependent negative effect on chondrogenesis with reduction of anabolic factors, elevation of catabolic enzymes, and increased inflammation. This effect was dependent on the cytokine used, with a stronger response in homologous use. A concentration of 1ng/mL IL-1β is sufficient to induce a significant inflammatory response of articular chondrocytes in a 3D pellet model. Standardization of the chondral inflammation model with a consequent detailed report of model characteristics is crucial for the comparability of different studies and the prediction of potential therapeutic consequences.

## Data Availability

The original contributions presented in the study are included in the article/Supplementary Material, further inquiries can be directed to the corresponding author.

## Supplementary Materials

The following supporting information can be downloaded at: https://www.mdpi.com/article/10.3390/cells14010030/s1, Figure S1: Negative controls of Caspase 3 immunohistochemistry of the bovine chondrocyte inflammation model with supplementation of IL-1β with different concentrations (0.1–100 ng/mL) and origin (human/bovine). Brown intracellular stain indicates apoptotic cells. Control was un-treated without cytokine stimulation. Scale bar 100 µm. IL-1β: interleukin-1β.
