# Supplementary material for: Comparison of Concentration- and Homology-Dependent Effects of the Proinflammatory Cytokine Interleukin-1β (IL-1β) in a Bovine Chondrocyte Inflammation Model
Source: Cells. 2024 Dec 31;14(1):30. doi: 10.3390/cells14010030 (PMC11719847; doi:10.3390/cells14010030)
Supplement: Supplementary file 1 [file cells-14-00030-s001.zip › cells-3403738-supplementary.pdf]

SUPPLEMENTARY MATERIAL

Supplementary Figure S1

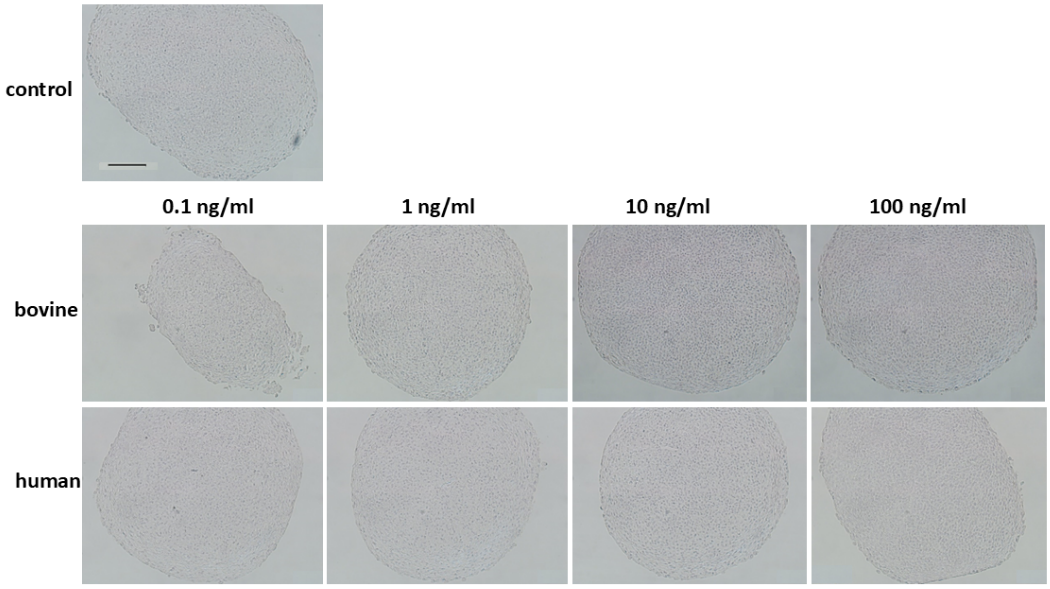

Supplementary Figure S1. Negative controls of Caspase 3 immunohistochemistry of the bovine chondrocyte inflammation model with supplementation of IL-1β with different concentrations (0.1–100 ng/mL) and origin (human/bovine). Brown intracellular stain indicates apoptotic cells. Control was un-treated without cytokine stimulation. Scale bar 100 μm. IL-1β: interleukin-1β.
